# Promoting Higher Quality Teacher–Child Relationships: The INSIGHTS Intervention in Rural Schools

**DOI:** 10.3390/ijerph17249371

**Published:** 2020-12-15

**Authors:** Kathleen Moritz Rudasill, Ray E. Reichenberg, Jungwon Eum, Jentry Stoneman Barrett, Yuenjung Joo, Emily Wilson, Martinique Sealy

**Affiliations:** 1 Virginia Commonwealth University, Richmond, VA 23284, USA; sealym@mymail.vcu.edu; 2 University of Nebraska-Lincoln, Lincoln, NE 68588, USA; rreichenberg@unl.edu (R.E.R.); jeum@unl.edu (J.E.); jbarrett3@unl.edu (J.S.B.); joo9240@huskers.unl.edu (Y.J.); emily.wilson@huskers.unl.edu (E.W.)

**Keywords:** teacher–child relationships, temperament, kindergarten, interventions, rural

## Abstract

Children’s relationships with teachers in kindergarten are crucial for academic and social success. Research shows that teacher–child relationships are predicated, in part, on children’s temperament. The “INSIGHTS into Children’s Temperament” intervention was intended to improve children’s and teachers’ understanding of their and others’ temperament, and has been shown to improve children’s social skills and self-regulation in urban, under-resourced schools. The current study is part of a replication of the effects of INSIGHTS with a sample in rural schools. The purpose was to test the effectiveness of INSIGHTS for promoting positive relationships between teachers and children in kindergarten. Two cohorts of kindergarten students (*N* = 127) and teachers (*N* = 30) were randomized into INSIGHTS or control conditions by school. Teachers reported on the quality of the teacher–child relationship before and after the INSIGHTS intervention (Time 1 and 2) using the Student–Teacher Relationship Scale: Short Form and provided a rating of children’s temperament with the Teacher School-Age Temperament Inventory at Time 1. Data were analyzed with hierarchical linear modeling. Two significant findings emerged. First, INSIGHTS promoted more closeness between teachers and children, regardless of temperament. Second, the INSIGHTS intervention was protective against the development of conflictual teacher–child relationships for children with negative reactivity.

## 1. Introduction

Children’s relationships with teachers are crucial for academic and social success in school [1,2,3,4,5]. Especially in the preschool and early elementary grades, these relationships set the trajectory for children’s experiences across the elementary school years and beyond [6,7,8]. Many factors affect the quality of this important relationship, and research points to child characteristics as paramount in teachers’ (and children’s) perceptions of teacher–child relationship quality [9,10,11]. Child factors such as temperament, behavior, socio-economic status, gender, and race have been implicated in teacher–child relationship quality [12,13,14,15]. There is less research about the role of teachers’ characteristics, but evidence suggests teacher efficacy, mental health, and personality play a role in teacher perceptions of their relationships with children [16,17,18]. Indeed, teachers establish the classroom milieu [19], especially in early childhood. Therefore, it is critical that teachers’ perceptions of children and their relationships with them are understood so that positive relationships may be nurtured.

In the current study, we investigate the effectiveness of the “INSIGHTS into Children’s Temperament” intervention (INSIGHTS) [20] for promoting positive teacher–child relationships between kindergarten teachers and children. Specifically, we ask whether the INSIGHTS intervention impacts teachers’ perceptions of the teacher–child relationship at the teacher level and at the child level. INSIGHTS is a universal social-emotional learning (SEL) intervention for kindergarten and first grade that includes parent, teacher, and classroom components. It is unique among SEL interventions because it is temperament-based; that is, INSIGHTS uses a temperament lens to instruct teachers and children about temperament and its role in children’s behaviors, and responses to interactions with others in the classroom and at home.

**Teacher–child relationships are important.** Teacher–child relationships are reliable predictors of children’s academic achievement and social and behavioral competence [21,22,23]. Positive teacher–child relationships provide an emotionally secure context for children, which encourages them to explore classroom resources more actively [24]. Such relationships are typically conceptualized as high in closeness (the extent to which relationships are marked by mutual warmth and trust) and low in conflict (characterized by discord and tension). Negative relationships, by contrast, are conceptualized as being low in closeness and high in conflict. Ample research connects positive teacher–child relationships to positive academic, social, and behavioral outcomes for children in preschool and elementary grades [21,22,25]. Negative relationships, namely those indicated as high in conflict, have been implicated in children’s suboptimal outcomes, such as lower achievement, less engagement, and poorer classroom and peer behavior [21,25,26].

Teachers who perceive their relationships with their students as close are more likely to provide positive instructional, behavioral, and emotional support to children and to encourage them to develop social and regulatory skills [27,28]. Those emotionally supportive contexts provide children more opportunities to develop and practice social skills, and encourage them to actively engage in school activities and successfully adjust to the school environment [29]. Early teacher–child relationships appear to have a lasting effect, as some of the literature indicates that positive (or negative) relationships between teachers and children in preschool and the early elementary grades predict long-term outcomes [7,8,21,22].

**Child temperament and teacher–child relationships.** Children’s temperament has been consistently linked to teacher–child relationship quality. Temperament is conceptualized as variability in children’s reactivity and regulation; it forms the attentional, affective, and activational foundation of personality [30,31]. An individual’s temperamental reactivity is demonstrated by the strength and duration of response to environmental stimuli; more reactivity means faster, longer-lasting, and more intense responses. As an example, a child with more reactive tendencies may become easily frustrated and demonstrate feelings with boisterous, angry behavior. High reactivity may also appear as excitement about a holiday or a friend’s visit. Temperamental regulation, on the other hand, is an individual’s modulation of reactivity; regulation operates upon reactivity by controlling emotional and behavioral responses [31]. Highly regulated children are typically able to check impulsive or negative behavior and, instead, initiate desired, appropriate behavior. Regulation and reactivity work together to contribute to children’s behavior [32]. If a child is high in reactivity and low in regulation, for example, the child may find the classroom environment to be particularly challenging [32,33,34,35].

Certain reactive and regulatory temperament characteristics are especially salient in academic settings. These characteristics are connected to children’s skill and comfort with social interaction (e.g., shyness) [9]; a tendency toward negative feelings (i.e., negative reactivity) [36]; a high baseline activity level that results in demonstrated difficulty with sitting or standing still, as well as a tendency to run from place to place (i.e., motor activity); and the ability to focus attention and complete tasks (i.e., task persistence) [20,37]. Children displaying temperament characteristics indicative of higher reactivity and lower regulation (i.e., high maintenance or “difficult” temperament) often have difficulty in school and classroom environments because their behavior places more demands on teachers and peers [38]. They are typically rated as having less closeness and more conflict with teachers [39]. Likewise, children who are more reactive but highly regulated (i.e., shy and withdrawn) are often rated as low in conflict and low in closeness [7]. Generally, high levels of regulation are rewarded in the classroom setting. More regulated children have an easier time with basic classroom expectations, such as sitting still, taking turns, remaining quiet, and following directions [35]; they are also advantaged during instructional activities, showing the ability to persist with difficult tasks, work in distracting environments, and pay attention to teacher instructions, resulting in better academic outcomes [33]. As such, more regulated children tend to be perceived by teachers as having more positive teacher–child relationships—those high in closeness and low in conflict [13].

**Goodness-of-fit in the classroom.** Children’s outcomes are a result of the extent to which the environment is a good (or poor) fit with their temperament. Classroom and school supports, such as positive student–teacher relationships, can be protective for children with temperament traits that make academic and social demands more challenging [40,41]. At the same time, deleterious aspects of classrooms and schools (e.g., teacher–child conflict) can exacerbate children’s negative temperament traits, thus cultivating suboptimal outcomes [39,42]. Thus, the classroom environment is a critical point of intervention for cultivating children’s success in school [38,43]. INSIGHTS, as a classroom-based intervention that provides children and teachers with temperament-based strategies for understanding and managing students’ emotion and behavior, was shown to be effective for improving classroom quality, particularly for children with more challenging temperaments (e.g., high-maintenance temperament) [3] in urban, high-need schools. In addition, results showed that INSIGHTS improved the teacher–child relationship [3].

**INSIGHTS into Children’s Temperament** (**INSIGHTS**)**.** INSIGHTS aims to optimize fit between children and classrooms by boosting teachers’ and children’s knowledge about individual differences in temperament, fostering positive interactions between children, promoting children’s emotional and behavioral regulation through awareness of their own and others’ temperament, and giving teachers temperament-based strategies for helping children manage their behavior and emotions. Such strategies increase teachers’ skills and improve strategies for interacting with and responding to children’s behavior [20]. The children’s portion of the intervention is conducted in classrooms for approximately 30 min every week; facilitators work with the classroom teacher to deliver instruction to children using puppets, videos, and vignettes. Teachers are provided with the INSIGHTS content during training sessions and coaching prior to sessions with children. With new skills, strategies, and awareness based on a temperament framework, we expect that teachers will have more positive perceptions of children which will, in turn, result in higher quality relationships. Similarly, we expect that children will benefit from their own understanding of temperament and from teachers’ improved responses, and that these changes will also result in more positive teacher–child relationships. Indeed, in a randomized, controlled study of INSIGHTS in a major US city with 22 high needs schools, McCormick et al. [3] found that children in treatment classrooms had improved teacher–child relationships after the intervention period. However, in that study, the researchers examined conflict and closeness as an aggregated variable, rather than as separate variables. In the present study, we add to the literature with an examination of the effectiveness of INSIGHTS for both teacher–child conflict and closeness, among teachers and children in rural schools.

**Current study.** The current study is part of a randomized control trial (RCT) to examine the effectiveness of the INSIGHTS intervention on children’s social and academic outcomes. Here, we investigated whether INSIGHTS would promote more positive relationships between teachers and children during kindergarten in rural schools. We were particularly interested in the extent to which INSIGHTS was not only promotive, but also protective for children depending on their temperament characteristics. Specifically, we used an intent-to-treat approach to investigate the moderating effect of INSIGHTS between children’s negative reactivity, motor activity, approach/withdrawal, and task persistence, and their closeness and conflict in relationships with kindergarten teachers. We expected that not only would INSIGHTS promote more closeness and less conflict for all children, but that INSIGHTS would be particularly beneficial for fostering more closeness and less conflict for children with higher levels of negative reactivity, motor activity, and withdrawal, and lower levels of task persistence.

## 2. Materials and Methods

**Participants and setting.** This study was approved by the Institutional Review Board at the University of Nebraska-Lincoln (#20180818622EP). Participants included 127 kindergarten students and 30 kindergarten teachers from 18 schools in two study cohorts. Schools were randomly assigned to treatment (INSIGHTS) or control (business as usual) groups. By school enrollment, students were then assigned to either be part of the treatment or control group (i.e., 60 students in the treatment group; 67 students in the control group). An average of 6.7 participating children per treatment classroom (range 3 to 10), and an average of 7.4 participating children per control classroom (range 4 to 11) had consent to participate. Although the INSIGHTS curriculum was presented to entire kindergarten classes, only participant data are reported. Teachers and children in this study live in rural areas within one Midwestern state. The rural school districts serve economically diverse populations; such socio-economic diversity is representative of school districts outside of urban areas within the Midwestern US. Teachers and parents provided consent to participate (and for children to participate), and children provided verbal assent prior to data collection.

Demographic data were collected from participants through online surveys of parents and teachers. According to parent report, a large majority (96%) of participating children were White, non-Hispanic, 42% were female, and 27% qualified for free or reduced-price school lunch. According to kindergarten teacher report, 99% of teachers were White, non-Hispanic; 97% were female, 100% had bachelor’s degrees, and 40% had master’s degrees. Teachers were between 26 and 58 years of age (*M* = 41.5) and had an average of 13 years of teaching experience.

**Recruitment, randomization, and timeline.** Participants were recruited by contacting schools, school districts, and educational service units (ESUs; units serving multiple school districts across a geographic area in the state) during the fall semesters of kindergarten (2018 and 2019 for cohorts 1 and 2, respectively). Once a school agreed to participate, we scheduled a meeting to talk with the principals, and kindergarten and first-grade teacher(s) in the schools to gain their consent to participate in the study. Teachers then assisted with recruiting parents by sending information home to families. Parents also received a digital version of the recruitment information.

In January of the kindergarten year, all participating teachers and children completed baseline (Time 1) measures; after baseline data were collected, schools were randomized into treatment (INSIGHTS) and control (i.e., business as usual) conditions [44]. The treatment schools were administered the INSIGHTS intervention over 10 weeks from late January through early March of 2019 or 2020, depending on the cohort, and then Time 2 data were collected from all treatment and control teachers and students in late April and May. Time 2 data collection for the second cohort (April–May 2020) occurred right after COVID-19 school closures. However, school closures did not disrupt the collection of time 2 teacher–child relationship quality data.

### 2.1. Measures

**Teacher–child relationship quality.** To measure teachers’ perceptions of the quality of their relationships with participating children, the Student–Teacher Relationship Scale-Short Form (STRS) was used [45]. The STRS contains 15 items of the original 28 items and uses a Likert-type format. There are two subscales for this measure: (1) closeness and (2) conflict. The closeness subscale consists of eight items and measures teachers’ perceptions of warmth and bonding with a particular child (e.g., *“I share an affectionate, warm relationship with this child”*). The conflict subscale consists of seven items and measures teachers’ perceptions of discord with a particular child (e.g., “*This child and I always seem to be struggling with each other*”). Teachers were asked to indicate how much each statement applies to their relationship with the student using a 5-point scale (definitely does not apply = 1, definitely applies = 5), with higher scores indicative of more closeness or conflict. Internal reliability with the current sample was good. In this study, Cronbach’s alphas for the STRS measured at Times 1 and 2 were as follows: closeness, α = 0.82; conflict α = 0.93. This is consistent with Cronbach’s alphas found in other studies [3]. The STRS-Short Form is widely used to measure teacher–child relationship quality and has robust support for the validity of scores [46].

**Child temperament.** To measure child temperament, the Teacher School-Age Temperament Inventory (T-SATI) was given to teachers at Time 1 [47]. The T-SATI contains 33 items and uses a Likert-type format. There are four temperament dimensions for this measure: (1) negative reactivity, (2) task persistence, (3) approach/withdrawal, and (4) motor activity. The first dimension, negative reactivity, consists of 11 items and measures the frequency and intensity with which a child expresses negative emotion (e.g., “*Gets upset when he/she can’t find something*”). The second dimension, task persistence, consists of nine items and measures the degree of self-direction that a child shows in completing tasks or responsibilities (e.g., “*Remembers to do assignments without being reminded*”). The third dimension, approach/withdrawal, consists of eight items and measures a child’s initial reaction to new situations or people (e.g., “*Is shy with adults he/she doesn’t know*”). The fourth dimension, motor activity, consists of five items and measures a child’s large body movement or motor activity level (e.g., “*Runs to get where he/she wants to go*”). Teachers were asked to indicate how often their student’s behavior is like the behavior described in each statement using a 5-point scale (never = 1, always = 5). Higher scores indicate more of the temperament dimension. The T-SATI demonstrated good internal reliability [47]. In the current study, Cronbach’s alphas for the T-SATI measured at Time 1 were negative reactivity: α = 0.95; task persistence: α = 0.94; approach/withdrawal: α = 0.94; motor activity: α = 0.90.

### 2.2. Intervention Procedures

**Facilitator training.** One facilitator conducted the intervention for Cohort 1 (four schools, seven classrooms) and a second facilitator was added to conduct the intervention for Cohort 2 (one facilitator was assigned to three schools and four classrooms; the second facilitator was assigned to three schools and three classrooms). Both facilitators are former classroom teachers and were trained to conduct the intervention by the developer of INSIGHTS, Sandee McClowry, Ph.D, RN, FAAN.

**Program delivery.** INSIGHTS was delivered to kindergarten teachers, their students, and participating parents. Teachers received professional development training (6 h on two Saturdays for a total of 12 h) from Dr. McClowry, then they partnered with their INSIGHTS facilitators to deliver the children’s program to their kindergarten classrooms. Once a week during the 10 weeks of the INSIGHTS classroom implementation, facilitators first met with teachers in their school as a group (if more than one grade-level teacher was participating at the school) for 30 min (for a total of an additional 5 h of training). Next, the facilitators worked with teachers to implement the 30 min intervention in each teacher’s classroom. Teachers were asked to use strategies and ideas from INSIGHTS with children during the week as they work with children to solve dilemmas (e.g., children having difficulty sharing materials, a fieldtrip is cancelled). Children were given classroom sets of puppets to work on solving dilemmas together. Parents also received training on INSIGHTS from facilitators and used the strategies at home with children (15 h of training conducted separately).

**Fidelity.** Fidelity was measured in several ways. Classroom sessions were recorded and 20% of them were reviewed by two members of the project staff against a fidelity checklist. Fidelity between facilitators was also measured. Teachers were also asked to rate the effectiveness of their facilitator each week (4.5 out of 5), and whether the facilitator used the materials to effectively teach the lesson (4.66 out of 5).

**Dosage.** To make comparisons easier between INSIGHTS and other interventions, we report dosage as suggested by Voils et al. [48] by including duration (“amount of time over which the intervention is intended to be administered” p. 2), frequency (“how often contact is intended to be made with participants per unit of time” p. 2), and amount (“length of each contact between the interventionist and the participant” p. 2). Voils and colleagues also suggested including intended dosage as a way to measure the difficulty of implementation. Table 1 shows our intended/actual duration, intended/actual frequency, and intended/actual amount for INSIGHTS. As shown in Table 2, participating children were in attendance 97% of the time on INSIGHTS days, and 84% of teachers completed the teacher professional development in person and 16% completed the professional development by watching pre-recorded sessions and completing quizzes and discussion questions.

**Data analysis.** Hierarchical regression was used to estimate the effect of the INSIGHTS intervention on the school-level Time 2 (post intervention) mean for teacher–child closeness and conflict, as well as the extent to which the relationship between the individual temperament dimension scores (i.e., negative reactivity, motor activity, task persistence, approach/withdrawal) and teacher–child relationship was moderated by participation in INSIGHTS. A total of eight, three-level (student/teacher/school) models were fit corresponding to the crossing of the four temperament dimensions with the two STRS domains (conflict, closeness). The cross-level interaction effect between intervention (school-level) and temperament dimension score (student-level) was specified as the regression of student-specific slope parameters (T-SATI dimension → STRS domain) on the binary experimental condition variable (INSIGHTS vs. control). All temperament dimension score variables were centered within context (CWC) [49]. Robust maximum likelihood estimation within the Mplus [50] environment was utilized for all models.

## 3. Results

**Figure 1:**
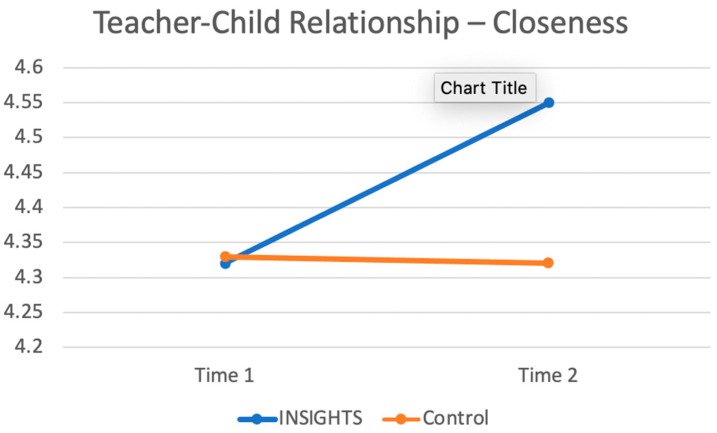
Pre- and post-INSIGHTS intervention of INSIGHTS and control groups.

**Descriptive statistics.** Independent samples *t*-tests were conducted to determine whether there was a statistically significant difference between INSIGHTS and control groups at baseline. Means, standard deviations, ranges, and *p*-values for teacher–child relationships and temperament at baseline (before the INSIGHTS intervention) are presented in Table 3. There were no significant differences between INSIGHTS and control groups at baseline. Paired samples *t*-tests were conducted to determine whether there was a statistically significant difference between the pre- and post- INSIGHTS intervention for the INSIGHTS and control groups. As shown in Table 4, differences in Time 1 and Time 2 means were statistically significant for teacher–child closeness for the INSIGHTS group (*t* = −4.737, df = 59, *p* < 0.001). After the INSIGHTS intervention, the INSIGHTS group teachers reported significantly more closeness compared to the control group teachers (Figure 1).

**Three-level closeness/conflict model.** First, an unconditional model was fit to determine the nature of the variability for the Time 2 teacher–child closeness and conflict scores across the three levels. These models yielded Level 2/Level 3 interclass correlation coefficient (ICC) estimates of 0.046/0.117 and 0.034/0.009 for the closeness and conflict models, respectively, suggesting minimal to moderate within-group homogeneity. Table 5 presents parameter estimates for the three effects of interest for the eight models considered in the current study. These effects included the autoregressive effect of Time 1 on Time 2 Closeness or Conflict (λ*_AR_*), the main effect of INSIGHTS on school-level average Time 2 Closeness or Conflict (λ*_treat_*), and interaction effects of INSIGHTS on the relationships between temperament dimension scores and Closeness or Conflict (λ*_int_*). Table 6 presents the simple slopes and intercepts describing the relationship between Temperament dimension score and Time 2 teacher–child closeness and conflict for the INSIGHTS and Control groups. As shown in Table 5, all eight models showed a strong relationship between the Time 1 and Time 2 outcomes. However, only teacher–child closeness had the main effect of INSIGHTS, and INSIGHTS was effective for all four temperaments (i.e., negative reactivity, task persistence, motor activity, approach/withdrawal). In addition, our results showed that the impact of children’s negative reactivity on teacher–child conflict was moderated by INSIGHTS. That is, children’s negative reactivity was not related to teacher–child conflict for the INSIGHTS group, but more negative reactivity predicted more teacher–child conflict at Time 2 for the Control group.

## 4. Discussion

Two main findings emerged from this study of the impact of INSIGHTS on teacher–child relationships for children with different temperaments. First, INSIGHTS promoted more closeness between teachers and children, regardless of temperament. That is, children in INSIGHTS classrooms had significantly higher closeness with teachers at Time 2 than children in control classrooms. Second, INSIGHTS moderated the effect of children’s negative reactivity on teacher–child conflict. Specifically, for children in INSIGHTS classrooms, negative reactivity was unrelated to teacher–child conflict whereas, in control classrooms, more negative reactivity predicted more conflict at Time 2. Each of these findings will be discussed in turn.

The INSIGHTS intervention promoted closeness between teachers and children from Time 1 (January of the kindergarten year) to Time 2 (March or April of the kindergarten year). This finding is congruent with both the tenets of INSIGHTS and results from the previous INSIGHTS intervention in urban, low resource schools [3]. INSIGHTS is premised on the notion that teachers will be better able to work with variability in students’ temperament-based behaviors if they understand temperament. The INSIGHTS intervention provides teachers with salient information about temperament, thus facilitating teachers’ empathy, understanding, and ability to work *with* children’s behavior, rather than turning to blame.

In the test of INSIGHTS conducted in an urban setting, McCormick et al. [3] found that children and teachers in INSIGHTS promoted better-quality relationships. In that study, teacher–child relationships were assessed as a total variable constituting positive relationship quality, such that a higher score indicated a better relationship. The focus of their study was not on the quality of the relationships, but rather on student engagement. McCormick et al. [3] found that children in INSIGHTS classrooms were more engaged in the school and academic activities, and that this was mediated, in part, by their positive teacher–child relationships. These findings are similar to other work pointing to the roles of teacher–child interactions and teacher self-efficacy in teacher–child relationship quality. Specifically, studies of teachers’ contributions to positive teacher–child relationships have shown that when teachers interact with students in ways that demonstrate interest in students’ emotions and activities, there is more closeness in the relationship [51,52]. Additionally, when teachers have more self-efficacy, especially for working with individual students, they are likely to feel more positively toward students and report more teacher–child closeness [53,54].

This also helps to explain the second main finding—that INSIGHTS was protective against conflict in the teacher–child relationship for children higher in negative reactivity. Thus, although INSIGHTS did not promote lower conflict for all children, it did serve to ameliorate the risk of conflict for children with higher levels of the negative reactivity. Research shows that children with temperament indicative of negative reactivity (e.g., “difficult” or “high maintenance”) tend to be viewed negatively by teachers or have more conflict and less closeness with teachers [38,55]. The INSIGHTS intervention teaches teachers to attribute children’s behaviors to inherent differences in temperament rather than to naughty behavior. In the previous efficacy trial of INSIGHTS that took place in under-resourced schools, urban schools, researchers found that children in INSIGHTS schools who had “high maintenance” temperaments (a combination of high negative reactivity, high motor activity, and low task persistence) displayed better classroom behavior and had higher engagement in classroom activities than their peers in the control classrooms, and these associations were partially explained by their relationships with teachers; that is, children in INSIGHTS classrooms with high-maintenance temperaments had more positive relationships with teachers and these, in turn, predicted better behavior and engagement [3].

Three limitations should be noted. First, there were only nine schools in each condition (INSIGHTS and control) which limited the power to detect effects. Second, there were two time-points in the current study; it would be helpful to see the longer-term benefits of INSIGHTS on children’s relationships with teachers. Third, teachers provided reports of both children’s temperament and their relationships with children. Although research suggests that teacher reports of children’s temperament is appropriate for the school environment [55], there is the risk of mono-method bias.

## 5. Conclusions

Results from this study from rural schools indicate that the INSIGHTS intervention promotes positive relationships between children and teachers in kindergarten. In addition, the intervention helps teachers understand children’s temperamental negative reactivity and, therefore, prevents the development of conflict in the teacher–child relationship. Finally, this study provides evidences for the generalizability of the intervention for use in diverse school contexts—urban and rural.

## Figures and Tables

**Table 1:** Intended/actual duration, frequency, and amount for INSIGHTS.

	Intended Duration	Actual Duration	Intended Frequency	Actual Frequency	Intended Amount	Actual Amount
Teacher PD	3 weeks	3 weeks	2 sessions	2 sessions	6 h per session; 12 h total	6 h per session; 12 h total
Teacher Coaching	10 weeks	10 weeks C1 9 weeks C2 ^2^	1× per week	1× per week	30 min per session; 5 h total	22.44 min per session; 3.74 h total
Classroom Lessons	10 weeks	10 weeks ^2^	1× per week	1× per week	30 min per session; 5 h total	30 min per session; 5 h total
Parent Lessons ^1^	10 weeks	10 weeks	Approx. every other week	Approx. every other week	2.5 h per session; 6 sessions total; 15 h	2.5 h per session; 6 sessions total; 15 h

Note: ^1^ Parent results are not reported in this manuscript, however we wanted to include parent dosage for informational purposes. ^2^ COVID-19-related school shutdowns prohibited our facilitators from attending the final week of the classroom intervention for Cohort 2. The classroom teachers taught the final week of INSIGHTS to their students. PD = Professional Development; C1 = Cohort 1; C2 = Cohort 2

**Table 2:** INSIGHTS intervention dosage.

	Teacher PD In-Person Attendance	Teacher PD Video Make-Up	Teacher Coaching Attendance	Student Attendance	Parent Session In-Person Attendance ^1^	Parent Session Video Make-Up ^2^
Cohort 1	96.88%	3.12%	97.88%	97.93%	90%	7.35%
Cohort 2	57.14%	42.86%	98.41%	95.02%	79.57%	15.59%

Note: ^1^ Parent results are not reported in this manuscript, however we wanted to include parent dosage for informational purposes ^2^ COVID-19 school shutdowns prohibited the last parent session in some schools during Cohort 2, and many parents watched the video to make-up for the missed class.

**Table 3:** Descriptive statistics for key variables at baseline (before INSIGHTS).

Variable	INSIGHTS (*N* = 60)		Control (*N* = 67)		*p*-Value
	M (SD)	Range	M (SD)	Range	
*Teacher–child relationships*					
Closeness (1–5)	4.32 (0.62)	2.5–5	4.33 (0.56)	2.88–5	0.916
Conflict (1–5)	1.87 (1.05)	1–4.86	1.79 (0.96)	1–4.86	0.635
*Child temperament*					
Negative reactivity (1–5)	1.92 (0.85)	1–4.45	1.88 (0.86)	1–4.64	0.795
Task persistence (1–5)	3.91 (0.77)	2.11–5	3.98 (0.78)	1.22–5	0.613
Withdrawal (1–5)	2.60 (0.91)	1.13–4.5	2.83 (0.88)	1.25–5	0.154
Motor activity (1–5)	2.33 (0.94)	1–4.6	2.12 (0.85)	1–4.4	0.180

Note: M = Mean; SD = Standard Deviation.

**Table 4:** Pre- and post-INSIGHTS intervention descriptive statistics for teacher–child relationships.

Variable	INSIGHTS (*N* = 60)			Control (*N* = 67)		
	M (*SD*)		*p*-Value	M (*SD*)		*p*-Value
	Time 1	Time 2		Time 1	Time 2	
Closeness	4.32 (0.62)	4.55 (0.55)	0.000	4.33 (0.56)	4.32 (0.57)	0.799
Conflict	1.87 (1.05)	1.79 (1.00)	0.223	1.79 (0.96)	1.90 (1.05)	0.073

**Table 5:** Estimates of focal parameters for three-level regression models.

Model	λ*_AR_*	λ*_treat_*	λ*_int_*
NR/Closeness	0.652 *	0.237 *	0.082
TP/Closeness	0.640 *	0.237 *	−0.024
AC/Closeness	0.641 *	0.237 *	0.067
AW/Closeness	0.627 *	0.238 *	−0.054
NR/Conflict	0.849 *	−0.176	−0.202 *
TP/Conflict	0.854 *	−0.175	−0.052
AC/Conflict	0.848 *	−0.175	0.024
AW/Conflict	0.867 *	−0.176	−0.009

Note: * denotes *p* < 0.05; NR = negative reactivity, TP = task persistence, AC = motor activity, AW = approach/withdrawal.

**Table 6:** Simple slopes and intercepts for T-SATI domain score predicting STRS domain score by experimental condition.

Model	INSIGHTS (1)		Control (0)	
	Intercept	Slope	Intercept	Slope
NR/Closeness	1.743	0.073	1.506	−0.009
TP/Closeness	1.794	0.009	1.557	0.033
AC/Closeness	1.791	0.014	1.554	−0.054
AW/Closeness	1.853	−0.036	1.615	0.017
NR/Conflict	0.211	−0.082	0.387	0.120
TP/Conflict	0.204	−0.090	0.379	−0.038
AC/Conflict	0.214	0.099	0.389	0.075
AW/Conflict	0.179	−0.070	0.355	−0.061

Note: T-SATI = Teacher School-Age Temperament Inventory, STRS = Student-Teacher Relationship Scale; NR = negative reactivity, TP = task persistence, AC = motor activity, AW = approach/withdrawal.
