# Addendum: Rudasill, K.M.; et al. Promoting Higher Quality Teacher–Child Relationships: The INSIGHTS Intervention in Rural Schools. *Int. J. Environ. Res. Public Health* 2020, *17*, 9371

**DOI:** 10.3390/ijerph18073519

**Published:** 2021-03-29

**Authors:** Kathleen Moritz Rudasill, Ray E. Reichenberg, Jungwon Eum, Jentry Stoneman Barrett, Yuenjung Joo, Emily Wilson, Martinique Sealy

**Affiliations:** 1 Virginia Commonwealth University, Richmond, VA 23284, USA; sealym@mymail.vcu.edu; 2 University of Nebraska-Lincoln, Lincoln, NE 68588, USA; rreichenberg@unl.edu (R.E.R.); jeum@unl.edu (J.E.); jbarrett3@unl.edu (J.S.B.); joo9240@huskers.unl.edu (Y.J.); emily.wilson@huskers.unl.edu (E.W.)

The authors wish to make the following correction to this paper [1]:

The original version of our article (Rudasill, K.M.; et al. Promoting Higher Quality Teacher–Child Relationships: The INSIGHTS Intervention in Rural Schools. 2020, 17, 9371) did not include the complete funding acknowledgement. The authors wish to change the information in the Funding section from:

**Funding:** This research was funded by the United States Institute of Education Sciences, grant number R305A180290.

to:

**Funding:** The research reported here was supported by the Institute of Education Sciences, U.S. Department of Education, through grant R305A180290, to the University of Nebraska-Lincoln. The opinions expressed are those of the authors and do not represent views of the Institute or the U.S. Department of Education.

The authors apologize for any inconvenience.
